# Differences in efficacy found in animals between recombinant forms of erythropoietin will not necessarily translate into differences in humans

**DOI:** 10.1038/sj.bjc.6600503

**Published:** 2002-08-12

**Authors:** H Malonne

**Affiliations:** Laboratoire de Physiologie et de Pharmacologie Fondamentales, Institut de Pharmacie, Université Libre de Bruxelles, Campus Plaine CP 206-3, Boulevard du Triomphe, B-1050 Bruxelles, Belgium

## Abstract

doi:10.1038/sj.bjc.6600503 www.bjcancer.com

© (2002) Cancer Research UK

## Sir

In your supplemental issue for volume 84, dated April 2001, Egrie and Browne (2001) reported a compilation of studies on the characterisation of novel erythropoiesis stimulating protein (NESP). NESP is a mutated form of the native hormone, erythropoietin. In NESP, five amino acids have been mutated (i.e., Ala30Asn, His 32Thr, Pro87Val, Trp88Asn and Pro90Thr) in order to incorporate two additional N-linked carbohydrate chains for a total of five carbohydrate moieties. In contrast, human erythropoietin as well as its recombinant form, r-HuEPO, has three N-linked carbohydrate chains. For comparative studies with NESP, Egrie and Browne (2001) used r-HuEPO.

In this report, it is shown that by increasing the sialic acid residue content of erythropoietin, the serum half life of the recombinant molecule, NESP, was approximately three-fold longer than that of r-HuEPO, primarily due to a prolonged clearance from the circulation. However, features in common between the two molecules are their nonlinear pharmacokinetics and pharmacodynamics. An increase in dose is required for both molecules when switching from a thrice-weekly (TIW) to a once-weekly (QW) regimen. For example, NESP 1.25 μg kg^−1^ TIW (total weekly dose: 3.75 μg kg^−1^) had to be increased four-fold to 15 μg kg^−1^ QW in order to achieve a nearly identical biological response. For r-HuEPO, the authors claimed that a 15-fold increase in the weekly dose of r-HuEPO was needed to switch from the TIW to the QW regimen in the animal model tested. In light of these findings, the authors continue to state that NESP has a ‘greater’ efficiency than r-HuEPO due to its ‘greater’ potency. Since pharmacodynamic responses depend on dose, route of administration, and dosing regimen, it is apparent that additional experimental data would be needed to substantiate a statement regarding the ‘greater’ potency of NESP. In the animal model tested, the data presented were derived from doses administered intravenously, and no comparison was made with alternative delivery routes (e.g., subcutaneous). Moreover, these results in animals appear to be of no consequence, since in humans, a similar biological response for NESP was achieved with an optimal weekly dose administered either once or divided into three doses (Macdougall, 1998). Similarly for r-HuEPO, 150 IU kg^−1^ TIW (total weekly dose: 450 IU kg^−1^) and 600 IU kg^−1^ QW (approximately 40 000 IU QW) regimens have been shown to produce a similar pharmacodynamic response in the rise of haemoglobin (Cheung *et al*, 1998). Thus, with the major differences in the erythropoiesis and red blood cell lifespan between rodents and humans, one must be cautious in extrapolating the data of *in vivo* biologic activity based upon small animal models to humans.

It is also important to note that potency cannot be equated with efficacy. In another set of experiments, efficacy was investigated using equimolar doses (i.e., equivalent peptide mass) of r-HuEPO, an erythropoietin analogue containing four N-linked carbohydrates, and NESP. For example, the results of one of these experiments showed that the haematocrit in mice increased by 22.8±2.3, 28.1±6.8, and 33.9±3.4 points, respectively. The authors concluded that since the bulkiness of NESP lowers its affinity for the erythropoietin receptor, clearance (i.e., *in vivo* potency) was the primary determinant for the higher magnitude in efficacy. With due respect to the authors, your readership is misled by the assessment that NESP has an enhanced efficacy compared with r-HuEPO or the four N-linked chain analogue. It appears that the authors are remiss in not considering that an equimolar dose is not equivalent to an equipotent dose, and thus conclusions regarding efficacy cannot be made. The use of equipotent doses would have been the appropriate experimental design for testing efficacy.

NESP is a product of only five point mutations, and the readership should be aware that some of the point mutations introduced into the erythropoietin molecule for the consensus sequences of additional carbohydrate moieties are nonconservative, especially those at residues 87, 88, and 90. In the native molecule, these residues contain ring structures. Since the predicted structure of endogenous erythropoietin is comprised of four α helices with a common secondary structural motif and bundled into a tertiary fold (Wrighton *et al*, 1996), these three bulky and more rigid residues potentially promote major turns in the polypeptide backbone. Thus in the NESP molecule, replacing these bulky residues with smaller and/or less rigid amino acids, the structure may change not only with additional carbohydrate moieties but also with the structural motif of the polypeptide backbone. As noted by the authors, the carbohydrate moieties may mask the point mutations themselves from the immune system; however, changes in secondary structure downstream from the point mutations cannot be ruled out.

To date, human studies in a dialysis population have confirmed the pharmacokinetic differences in half life and clearance between NESP and r-HuEPO (Macdougall, 1999). However, studies in humans using NESP have shown an efficacy profile that is comparable to r-HuEPO (Coyne *et al*, 2000; Nissenson *et al*, 2000; Locatelli *et al*, 2001). These studies reinforce the fact that results garnered from animal models are not necessarily indicative of what is to be ascertained in humans.
